# HtrA-Dependent E-Cadherin Shedding Impairs the Epithelial Barrier Function in Primary Gastric Epithelial Cells and Gastric Organoids

**DOI:** 10.3390/ijms25137083

**Published:** 2024-06-27

**Authors:** Marina Canadas-Ortega, Iris Mühlbacher, Gernot Posselt, Sebastian Diechler, Christian Daniel Ferner, Francesco Boccellato, Oliver Owen Koch, Daniel Neureiter, Michael Weitzendorfer, Klaus Emmanuel, Silja Wessler

**Affiliations:** 1 Department of Biosciences and Medical Biology, Division of Microbial Infection and Cancer, Paris-Lodron University of Salzburg, 5020 Salzburg, Austria; marina.canadas-ortega@plus.ac.at (M.C.-O.); gernot.posselt@plus.ac.at (G.P.); sebastian.diechler@plus.ac.at (S.D.); christiandaniel.ferner@plus.ac.at (C.D.F.); 2 Cancer Cluster Salzburg, 5020 Salzburg, Austria; 3 Department of Surgery, Paracelsus Medical University, 5020 Salzburg, Austria; i.muehlbacher@salk.at (I.M.); o.koch@salk.at (O.O.K.); m.weitzendorfer@salk.at (M.W.); k.emmanuel@salk.at (K.E.); 4 Center for Tumor Biology and Immunology (CTBI), Paris-Lodron University of Salzburg, 5020 Salzburg, Austria; 5 Nuffield Department of Clinical Medicine, Ludwig Institute for Cancer Research, University of Oxford, Oxford OX37DQ, UK; francesco.boccellato@ludwig.ox.ac.uk; 6 Institute of Pathology, Cancer Cluster Salzburg, Paracelsus Medical University/University Hospital Salzburg (SALK), 5020 Salzburg, Austria; d.neureiter@salk.at

**Keywords:** *Helicobacter pylori*, HtrA, E-cadherin, CagA, organoids, mucosoids

## Abstract

Impaired E-cadherin (Cdh1) functions are closely associated with cellular dedifferentiation, infiltrative tumor growth and metastasis, particularly in gastric cancer. The class-I carcinogen *Helicobacter pylori* (*H. pylori*) colonizes gastric epithelial cells and induces Cdh1 shedding, which is primarily mediated by the secreted bacterial protease high temperature requirement A (HtrA). In this study, we used human primary epithelial cell lines derived from gastroids and mucosoids from different healthy donors to investigate HtrA-mediated Cdh1 cleavage and the subsequent impact on bacterial pathogenesis in a non-neoplastic context. We found a severe impairment of Cdh1 functions by HtrA-induced ectodomain cleavage in 2D primary cells and mucosoids. Since mucosoids exhibit an intact apico-basal polarity, we investigated bacterial transmigration across the monolayer, which was partially depolarized by HtrA, as indicated by microscopy, the analyses of the transepithelial electrical resistance (TEER) and colony forming unit (cfu) assays. Finally, we investigated CagA injection and observed efficient CagA translocation and tyrosine phosphorylation in 2D primary cells and, to a lesser extent, similar effects in mucosoids. In summary, HtrA is a crucially important factor promoting the multistep pathogenesis of *H. pylori* in non-transformed primary gastric epithelial cells and organoid-based epithelial models.

## 1. Introduction

*Helicobacter pylori* (*H. pylori*) is a bacterial carcinogen, which persistently infects the epithelial lining of the human stomach and induces several gastric disorders, ranging from chronic gastritis and peptic ulcers to lymphoma of the mucosa-associated lymphoid tissue (MALT) system and gastric cancer [1,2]. The induction and progression of pathogen-induced diseases require direct interaction of *H. pylori* with the gastric epithelium, which controls a complex network of signal transduction pathways resulting in a multistep pathogenesis [3].

A key molecule in homeostasis of gastric epithelial cells is the cell adhesion protein and tumor suppressor E-cadherin (Cdh1). Cdh1 establishes calcium-dependent homophilic interactions between its extracellular domains in *cis* and *trans* between neighboring epithelial cells ensuring intact cell-to-cell adhesions [4]. Cdh1 recruits β-catenin and p120 catenin to its intracellular domain, which stabilizes the adherens junction complex [5]. Generally, loss of Cdh1 functions can be caused by transcriptional downregulation through promoter hypermethylation or microRNA, loss-of-function mutations, or ectodomain shedding [6,7]. Cdh1 dysregulation is implicated in gastric tumorigenesis as well as in tumor progression, invasion, and metastasis and serves as a negative prognostic factor for gastric cancer [8], which is essentially integrated in the molecular classification of gastric cancer [9]. Improper Cdh1 function subsequently releases β-catenin and p120 catenin from the complex, which can translocate into the nucleus where they interfere with TCF/LEF1- and Kaiso-controlled gene transcription [10,11]. Especially in the intestinal type [12] and hereditary diffuse type of gastric cancer [13], the loss of proper Cdh1 functions are of major importance for the aggressiveness of the disease.

The release of the Cdh1 ectodomain was directly associated with *H. pylori* infections. *H. pylori* secretes the active serine protease high temperature requirement A (HtrA), which cleaves the extracellular domain of Cdh1 on the cell surface, resulting in a local opening of lateral cell-to-cell junctions [14]. Cdh1 was the first identified HtrA substrate; later, the desmosomal desmoglein-2 (Dsg-2) and the tight junction proteins occludin and claudin-8 were discovered as additional targets of *H. pylori* HtrA [15,16]. In fact, HtrA activity was found to be the main factor for the opening of lateral junctions between epithelial cells, while host-derived proteases, such as matrix metalloproteases or ADAM proteases only play a minor role in *H. pylori* infections [14,15]. HtrA-mediated Cdh1 shedding and the subsequent opening of cell–cell adhesions facilitate transmigration of *H. pylori* through the intercellular space [14] and the translocation of the virulence factor cytotoxin-associated gene A (CagA) via a type-IV secretion system (T4SS) into host cells [16]. Once CagA is injected into the cytosol, it becomes rapidly tyrosine-phosphorylated by Src family kinases (SFK) and c-Abl [17,18] and directly regulates a complex network of signaling pathways, leading to epithelial depolarization.

The functional interaction between *H. pylori* and gastric epithelial cells has been exclusively investigated in gastric epithelial cell lines originating from adenocarcinoma. However, these cell lines show significant changes in Cdh1 functions and non-receptor tyrosine kinase signaling due to neoplastic changes, leading to a loss of epithelial barrier function and cell polarity. Gastric mucosoids are recently established infection models for *H. pylori*, representing a stem cell-driven, highly polarized, columnar epithelium, which produces mucus on the apical side [19]. In this study, we used gastric mucosoids from healthy donors to investigate the influence of HtrA on *H. pylori* pathogenesis under non-transformed conditions.

## 2. Results

### 2.1. H. pylori HtrA Cleaves E-Cadherin on Primary Gastric Epithelial Cells

**Figure 1:**
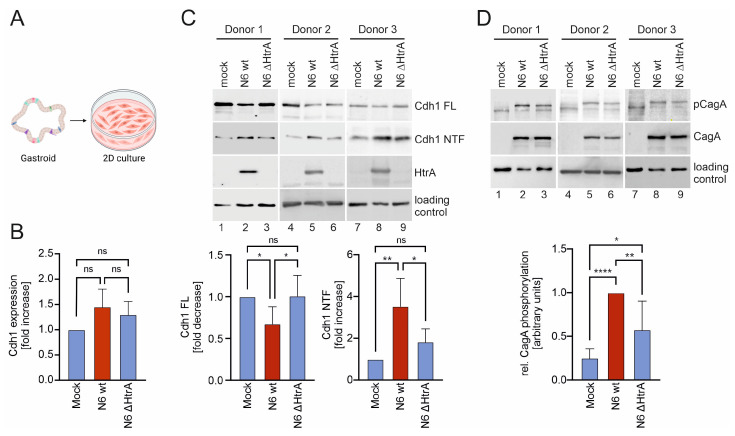
**HtrA-mediated cleavage of E-cadherin in organoid-derived primary cells.** (**A**) Scheme of human gastroid cultures. Primary gastric cells were isolated from gastroids and seeded for 2D culture in cell dishes. Created with BioRender.com. (**B**) 2D cell cultures were infected with *H. pylori* strains N6 wt and N6 ΔhtrA for 16 h at an MOI of 10 or left untreated (mock). Cdh1 expression was analyzed by real-time PCR from independent experiments (n = 3). Results are presented as fold increase with the levels of mock-treated cells set to 1. (**C**) Whole cell lysates and supernatants of infected 2D cultures from three different donors were analyzed by Western blot for full-length E-cadherin (Cdh1 FL) and soluble N-terminal cleavage fragments (Cdh1 NTF). HtrA was included as an infection control and GAPDH or β-actin were detected as loading controls (upper panels). The relative amounts of Cdh1 FL (n = 5) and soluble Cdh1 NTF (n = 3) were quantified by blot densitometry and normalized to the corresponding loading control. Results are presented as fold increase with the levels of mock-treated cells set to 1 (lower panels). (**D**) CagA translocation and phosphorylation were analyzed by Western blotting using antibodies against phospho-tyrosine (pCagA), CagA, and GAPDH or β-actin as loading controls (upper panels). pCagA was quantified by blot densitometry (n = 7) and normalized to the loading control (lower panel). ns, not significant; *, *p* ≤ 0.05; **, *p* ≤ 0.01; ****, *p* ≤ 0.0001.

Cdh1-positive gastric epithelial cells derived from gastric adenocarcinoma, such as MKN-28 or NCI-N87 cells have previously been used to study HtrA-mediated Cdh1 cleavage [14,15], while investigations utilizing healthy human epithelial cells are not yet available. Hence, human antral organoid cultures (gastroids) were generated from sleeve gastrectomy samples and transformed into 2-dimensional (2D) gastric epithelial cell monolayers (Figure 1A). To investigate Cdh1 functions, 2D cell cultures derived from different donors were infected with *H. pylori* wild type (wt), an isogenic *htrA* deletion mutant (∆htrA) [20], or left untreated (mock) for 16 h. Since loss of adhesion in many types of cancer is often attributed to decreased E-cadherin expression, Cdh1 expression was quantified by real-time PCR and showed a slight transcriptional upregulation in response to *H. pylori* infection (Figure 1B). Ectodomain shedding of Cdh1 in response to *H. pylori* HtrA was investigated in Western blot analyses through the detection of the loss of Cdh1 full-length (FL) protein and the increase in the soluble extracellular domain of Cdh1 (NTF) in the supernatant of infected cells. In three individual organoid lines, *H. pylori* wt induced a significant decrease in Cdh1 FL, which was not observed in infections with *H. pylori* ∆htrA. Correspondingly, the amounts of Cdh1 NTF in the supernatant of *H. pylori* wt-infected cells increased compared to supernatants of mock-treated cells or after infection with *H. pylori* ∆htrA (Figure 1C, upper panels). The signals for Cdh1 FL and Cdh1 NTF from independent experiments were quantified, demonstrating a significant HtrA-dependent Cdh1 cleavage on primary gastric epithelial cells (Figure 1C, lower panels). These data not only confirm the previous data obtained from gastric cancer cell lines [14,15,16], but also demonstrate stable Cdh1 expression and suggest a slight transcriptional increase upon *H. pylori* infection.

HtrA-mediated Cdh1 cleavage fosters CagA translocation via the T4SS into host cells [16]. To investigate CagA injection, primary gastric epithelial cells were grown to confluence and infected with *H. pylori* wt or the *H. pylori* ∆htrA deletion mutant. CagA was efficiently translocated and phosphorylated in *H. pylori* wt-infected primary epithelial cells as monitored by the detection of tyrosine-phosphorylated CagA (pCagA), whereas the HtrA-negative deletion mutant transported only minor amounts of CagA into primary epithelial cells (Figure 1D, upper panels). This underlines that HtrA is important for CagA translocation in gastric epithelial cells, which was further supported by the quantification of independent experiments with different donors, demonstrating the significance of HtrA in the process of CagA delivery (Figure 1D, lower panel).

### 2.2. HtrA-Mediated Cdh1 Shedding Facilitates CagA Translocation into Polarized Mucosoid Cultures

**Figure 2:**
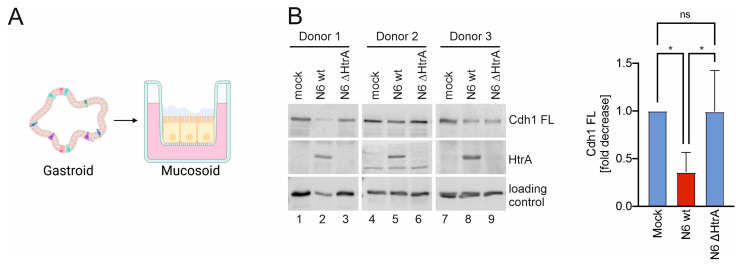
***H. pylori* cleaves E-cadherin in polarized mucosoid cultures.** (**A**) Cells obtained from gastroids were cultured in 0.4 µm transwell filters to form a columnar monolayer with apical mucus production. Created with BioRender.com. (**B**) Mucosoids were infected with *H. pylori* strains N6 wt, N6 ΔHtrA for 40 h at MOI 10 or left uninfected. Whole cell lysates were analyzed by Western blotting to detect full-length E-cadherin (Cdh1 FL) and HtrA. GAPDH or β-actin were used as loading controls (left panel). The relative amount of Cdh1 FL (n = 3) was quantified by blot densitometry and normalized to the corresponding loading control. Results are presented as fold decrease with the levels of mock-treated cells set to 1 (right panel). ns, not significant; *, *p* ≤ 0.05.

Mucosoid cultures have recently been established as an advanced infection model for *H. pylori*, which has many advantages over spherical organoids with basal-out architecture or 2D cultures [19]. Gastric mucosoids are air–liquid interface (ALI) cultures of gastroids grown on transwell filters, which produce considerable amounts of mucus on the apical surface of a highly polarized columnar epithelial monolayer (Figure 2A). Since mucosoid cultures mimic the gastric epithelium particularly well, Cdh1 cleavage was investigated in mucosoids from different donors after infection with *H. pylori* wt and *H. pylori* ∆htrA. In all tested donor lines, *H. pylori* wt decreased Cdh1 FL in whole cell lysates significantly compared to *H. pylori* ∆htrA and uninfected cells (Figure 2B, left panel). Cdh1 NTF could neither be detected in the basal medium nor, due to technical reasons, in the viscous mucus supernatant of the ALI cultures by means of Western blotting. Quantification of Cdh1 FL from independent experiments with different donors revealed a significant HtrA-dependent loss of Cdh1 FL in mucosoid cultures (Figure 2B, right panel).

**Figure 3:**
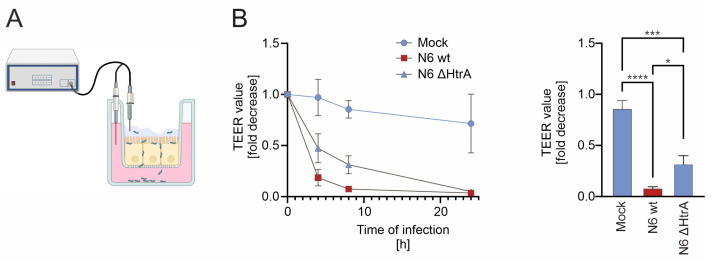
**HtrA impairs the epithelial junctional integrity.** (**A**) Cells were grown on 8 µm transwell filters for 21 days to establish a polarized monolayer allowing TEER measurement. Created with BioRender.com. (**B**) Mucosoids were mock-treated or infected with *H. pylori* N6 wt and N6 ΔhtrA. TEER was measured after 4, 8, and 24 h (n = 3). HtrA-dependent differences in TEER were analyzed after 8 h. Data were correlated to TEER value after 0 h and expressed as fold decrease (right panel). ns, not significant; *, *p* ≤ 0.05; ***, *p* ≤ 0.001; ****, *p* ≤ 0.0001.

The degree of cellular polarization of an epithelium depends on the functionality of the intercellular adhesions, especially the tight junctions, which can be investigated by transepithelial electrical resistance (TEER) measurement (Figure 3A). Mucosoids form a highly polarized cell monolayer, presenting an intact epithelial barrier. Infections with *H. pylori* wt led to a strong decrease in TEER, which was significantly weaker after infection with *H. pylori* ∆htrA within the first 8 h (Figure 3B, left and right panels). After 24 h of infection with both, *H. pylori* wt and ∆htrA did not show significant differences in TEER eventually due to severe damage of primary gastric epithelial cells independent of HtrA (Figure 3B, left panel).

**Figure 4:**
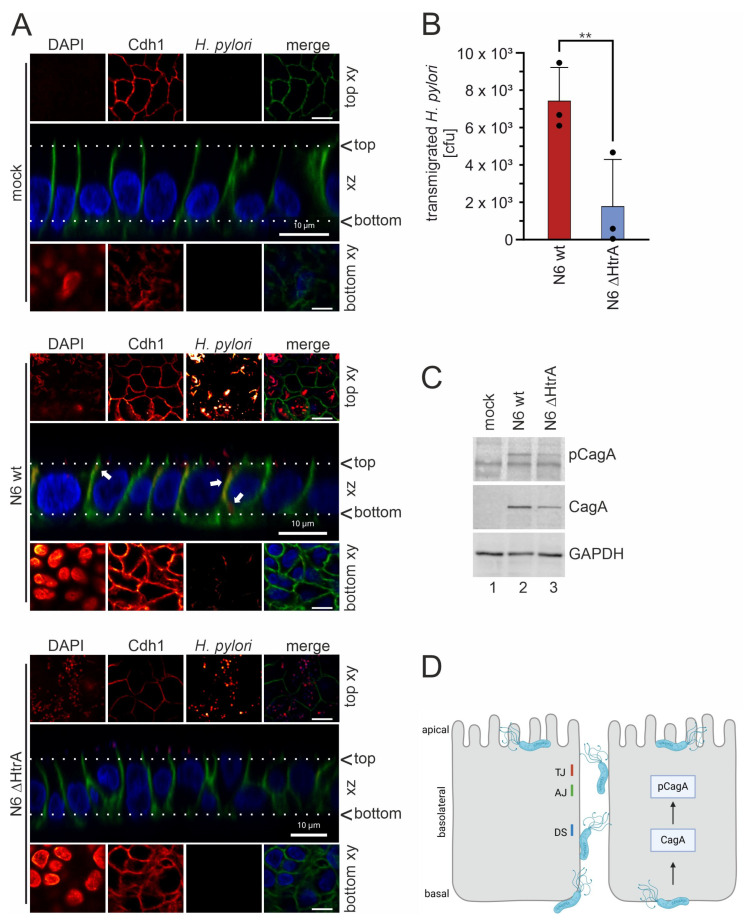
**HtrA is necessary for efficient epithelial transmigration of *H. pylori*.** (**A**) Cells were grown on 0.4 µm transwell filters for 21 days prior to infection and either left untreated (mock, upper panel) or infected with *H. pylori* wt (N6 wt, middle panel) or *H. pylori* ΔhtrA (N6 ΔHtrA, lower panel). Of all samples, random optical *xz* sections were generated (central *xz* images) and at the height of the white dotted lines, top *xy* and bottom *xy* images were acquired to analyze surface colonization (top *xy*) and basally localized bacteria (bottom *xy*). Chanel intensities (glow) are shown for *xy* DAPI (blue), Cdh1 (green), and *H. pylori* (red) with false color overlays for *xy* merge and *xz* sections. Scale bars, 10 µm. (**B**) Cells were grown on 8 µm transwell filters for 21 days to establish a polarized monolayer to analyze transmigration. Quantification of transmigrated *H. pylori* after infection for 8 h with N6 wt and N6 ΔhtrA in cfu assays (n = 3). (**C**) Representative whole cell lysates from mucosoids grown on 0.4 µm filters were analyzed in Western blots to detect pCagA, CagA, and GAPDH. (**D**) Model of the *H. pylori* multistep pathogenesis, which involves HtrA-mediated opening of tight junctions (TJ), adherens junctions (AJ), and desmosomes (DS) prior to CagA translocation at the basolateral site. Created with BioRender.com. **, *p* ≤ 0.01.

The opening of lateral cell junctions is linked to an enhanced paracellular transmigration of *H. pylori* and is accompanied by cellular depolarization [14]. To further examine the epithelial barrier integrity, we analyzed the transmigration of *H. pylori* across the polarized epithelial cell layer. To monitor bacterial localization by confocal laser scanning microscopy, mucosoids were left untreated or infected with either *H. pylori* wt or *H. pylori* ∆htrA. Of note, uninfected mucosoids form a tall columnar epithelium with a height of 14–17 µm, whereas infection with *H. pylori* wt and to a lesser extent *H. pylori* ∆htrA reduced the epithelial height to 8–10 µm and 10–12 µm, respectively (Figure 4A, central *xz* sections in the upper, middle, and lower panels). *H. pylori* wt and *H. pylori* ∆htrA were both able to pass the dense mucus layer to a similar extent and both established a robust surface colonization (Figure 4A, top *xy* and sections of middle and lower panels). Whilst *H. pylori* wt entered the intercellular space (Figure 4A, *xz* section of middle panel, white arrows) and localized at the basolateral side (Figure 4A, bottom *xy* of middle panel), *H. pylori* ∆htrA was not detected at the basolateral or basal membrane domains (Figure 4A, lower panel). In order to quantify the number of transmigrating bacteria, we performed cfu (colony forming unit) assays using the medium in the lower reservoir of the transwell filter TEER experiments. While *H. pylori* wt efficiently transmigrated through the polarized monolayer, there was a drastic decrease in *H. pylori* transmigration to the basal reservoir after deletion of *htrA* (Figure 4B). Finally, *H. pylori* wt effectively delivered CagA into mucosoids, which is consistent with a previous report [19]. Here, we observed that the *H. pylori* ∆htrA showed a decrease in CagA translocation (Figure 4C). In summary, we have shown that HtrA-mediated Cdh1 shedding is implicated in *H. pylori* pathogenesis to underline the important HtrA effects on non-transformed human primary gastric epithelial cells.

## 3. Discussion

The investigation of *H. pylori* HtrA has been intensified over the last 15 years since it became clear that HtrA is a key factor in both the physiology and pathogenesis of *H. pylori*. HtrA is a widely expressed bacterial chaperone and serine protease. However, in contrast to many other organisms, HtrA is absolutely essential for *H. pylori* survival [21,22,23]. A genomic *htrA* deletion mutant in *H. pylori* could not be generated for a long time [14,21] and natural HtrA-negative *H. pylori* isolates are unknown [22]. Exclusively in *H. pylori* N6, the *htrA* gene deletion was rendered possible alongside a random mutation in the *secA* gene [20]. Studies with selective HtrA inhibitors and the availability of an *H. pylori* ∆htrA deletion mutant yielded the final proof of the key impact of HtrA on *H. pylori* pathogenesis [14]. The implication of CagA in *H. pylori*-induced gastric diseases is well established; however, the activity of HtrA seems to be decisive. Recently, a single-nucleotide polymorphism (SNP) in the HtrA protein (L171) was identified [24,25], which leads to increased stability of active HtrA [26]. The L171 SNP strongly correlates with the development of gastric cancer [24,25]. Therefore, we conclude that the functional interaction between HtrA and CagA determines the extent of *H. pylori* disease.

The finding that HtrA-mediated Cdh1 cleavage facilitates bacterial transmigration and translocation of CagA is remarkable and in this study, we have shown that HtrA (i) mediates Cdh1 cleavage on the surface of highly polarized gastric epithelial cells and (ii) enables CagA injection and phosphorylation in the cytosol of non-transformed healthy cells without derailed tyrosine kinases.

We detected significant HtrA-mediated Cdh1 cleavage, transmigration, and CagA delivery in primary gastric epithelial cells and highly polarized mucosoids. Previously used gastric tumor cell lines often express aberrantly deregulated host proteases. A number of Cdh1-cleaving host proteases are known, including matrix metalloproteases (e.g., MMP-3, MMP-7, or MMP-9), A Disintegrin and metalloproteinase domain-containing (ADAM) proteins (e.g., ADAM-10 and ADAM-15), kallikreins, and further, which are often upregulated or constitutively activated in *H. pylori*-associated gastritis and tumors [27]. Therefore, soluble Cdh1 NTF serves as an important biomarker for many types of cancer and predicts a higher aggressiveness of tumors [28]. Even though we have not analyzed the activity of host proteases in mucosoids of healthy donors in this study, we assume that these host proteases play only a minor role since inhibition of MMPs and ADAM proteases revealed that *H. pylori* HtrA is the main protease targeting Cdh1 during infection [14,15]. In particular, the quantification of Cdh1 cleavage in our report demonstrates the important role of HtrA activity in *H. pylori* pathogenesis.

Generally, Cdh1 expression on the surface of epithelial cells is highly dynamic and dysfunctional cadherin molecules are rapidly internalized and removed via the 26S proteasome system [29]. Therefore, cells immediately re-express Cdh1 to re-establish intercellular adhesions. This fits with the observation that HtrA-mediated Cdh1 cleavage is a local process, which occurs in close proximity to *H. pylori*, leading to local opening of intercellular adhesions [30]. In fact, in human biopsies, *H. pylori* was found in the intercellular space below the lateral junctions, which have already closed again [16,31]. This phenomenon requires Cdh1 re-expression. Indeed, we observe a slight increase in Cdh1 mRNA expression after *H. pylori* infections, which subsequently counteracts the decrease in Cdh1 protein in primary gastric epithelial cells.

CagA injection and phosphorylation are clearly facilitated by HtrA-mediated opening of intercellular adhesions. We have shown that in early phases of infections, HtrA is implicated in the disruption of the epithelial barrier function as monitored by the decrease in TEER, which is likely induced by the HtrA-mediated cleavage of the tight junction proteins occludin and claudin-8 [16]. HtrA-mediated Cdh1 and Dsg-2 shedding also contribute to the loss of TEER since dysfunction of adherence junctions, desmosomes, and tight junctions are closely interconnected [32,33]. According to the multistep pathogenesis model (Figure 4D), transmigrating *H. pylori* can attach to apical, basolateral, and basal membranes of the polarized gastric epithelium. Both apical CEACAM and basolateral/basal β1-integrin receptors have been identified as key molecules necessary for CagA injection [34,35,36]. However, the detailed molecular injection mechanism is still not completely understood, but confocal microcopy and T4SS pilus expression data have suggested that CagA translocation occurs at basolateral and basal membranes co-localizing with β1-integrin expression [16,34]. Hence, HtrA-mediated opening of intercellular adhesions and bacterial transmigration results in an increase in CagA delivery into primary cells of mucosoid cultures.

## 4. Conclusions

The interaction between *H. pylori* and gastric epithelial cells is critical for the induction and progression of gastric disorders. In vivo studies of HtrA function in animals are not possible due to the lack of a rodent-adapted *∆htrA* knockout *H. pylori* strain. Hence, the establishment of primary epithelial models based on organoids and 2D mucosoids represents an important step forward in understanding *H. pylori*-mediated pathogenesis. However, organoid- and mucosoid-based models also have limitations, as they do not represent the full complexity of native tissues and microenvironment, lacking the stroma or components of the immune, vascular, and nervous system. Despite these limitations, organoids remain a powerful tool in *H. pylori* research, providing valuable insights and offering a more physiological model than tumor cells.

In our study, we have highlighted the critical role of HtrA in *H. pylori* pathogenesis. We have demonstrated that HtrA mediates Cdh1 cleavage on the surface of highly polarized gastric epithelial cells, enabling CagA injection and phosphorylation in the cytosol of non-transformed healthy cells. HtrA-mediated disruption of epithelial barrier function further supports the pathogen’s ability to promote infection. Overall, the interaction between HtrA and CagA is crucial for the severity and progression of *H. pylori*-associated disease. Based on these findings, pharmacological inhibition of HtrA is a desirable goal because (i) HtrA is a druggable target and (ii) it is an essential protein in bacterial physiology. This could assist antibiotic therapy or, in the case of treatment of multi-resistant *H. pylori* strains, potentially eliminate the infection.

## 5. Materials and Methods

### 5.1. Gastric Organoids and Mucosoids

Tissue samples from six individuals (3 female and 3 male patients with an average age of 32.2 ± 9.4 years and with an pre-operative average body mass index [kg/m^2^] of 46.8± 7.1; for more details, see Table 1) who underwent gastric sleeve resection at the Department of Surgery were prepared at the Institute of Pathology at the Paracelsus Medical University and University Hospital Salzburg under sterile conditions to avoid any bacterial contamination. Subsequently, all samples were subjected to standardized pathological processing to exclude *H. pylori* gastritis.

Mucosoids were cultured as described previously [19]. An amount of 2 × 10^5^ antral organoid-derived primary gastric cells were seeded on collagen-coated 0.4 µm transwell filters (Sigma-Aldrich, Vienna, Austria) and cultured for 2 to 3 weeks in organoid medium composed of 18.5% *v/v* Advanced DMEM/F12 (Thermo Fischer, Vienna, Austria), 50% *v/v* Wnt3A conditioned medium, 25% *v/v* R-spondin-1 conditioned medium, 10 mM 4-(2-hydroxyethyl)-1-piperazineethanesulfonic acid (Carl Roth, Karlsruhe, Germany), 2 mM L-glutamine (Biowest, Nuaillé, France), 2% *v/v* B27 (Thermo Fischer, Vienna, Austria), 1% *v/v* N2 (Thermo Fischer, Vienna, Austria), 20 ng/mL human EGF (Thermo Fischer, Vienna, Austria), 150 ng/mL human noggin (Peprotech, London, UK), 150 ng human FGF-10/mL (Peprotech, London, UK), 10 mM nicotinamide (Sigma-Aldrich, Vienna, Austria), 10 nM human gastrin (Sigma-Aldrich, Vienna, Austria), 1 μM A83–01 (Sigma-Aldrich, Vienna, Austria), and 7.5 μM Y-27632 (Sigma-Aldrich, Vienna, Austria). For 2D cultures, 2.5 × 10^5^ mucosoid-derived primary gastric cells were seeded on collagen-coated 24-well plates (Greiner, Kremsmünster, Austria) and cultured overnight in 2D medium containing 88% *v/v* Advanced DMEM/F12 (Thermo Fischer, Vienna, Austria), 10 mM 4-(2-hydroxyethyl)-1-piperazineethanesulfonic acid (Carl Roth, Karlsruhe, Germany), 2 mM L-glutamine (Biowest, Nuaillé, France), and 10% *v/v* FBS (Th. Geyer, Renningen, Germany).

### 5.2. Bacteria and Infection Experiments

*H. pylori* strains N6 wild type (wt) and N6 ∆htrA [20] were cultivated on agar plates containing 10% horse serum (Th. Geyer, Renningen, Germany) under microaerophilic conditions and 37 °C for 24 h. One hour prior to infection of 2D cultures, the medium was exchanged to serum-free media. Infections with *H. pylori* were carried out for 16–20 h at an MOI of 10. Mucosoids were apically infected with *H. pylori* for 48 h at an MOI of 10 after removal of the mucus.

### 5.3. SDS-PAGE and Western Blot

Cells were harvested in lysis buffer (20 mM Tris pH 7.5, 1 mM EDTA, 100 mM NaCl, 1% Triton X-100, 0.5% DOC, 0.1% SDS supplemented with 1× PIT (protease inhibitor, Roche, Basel, Switzerland), 20 mM β-glycerophosphate, 20 mM sodium fluoride, 1 mM sodium molybdate, and 1 mM sodium orthovanadate). Whole cell lysates were cleared from debris by centrifugation. Supernatants were collected for analyses of soluble proteins. Equal amounts of protein were separated by SDS-PAGE and blotted on nitrocellulose membrane (Lactan, Graz, Austria). The following antibodies were used: anti-human E-cadherin (Cdh1; Cell Signaling Technology, Leiden, The Netherlands), anti-human E-cadherin EC5 (Abcam, Cambridge, UK), phospho-tyrosine (4G10, Cell Signaling Technology, Leiden, The Netherlands), anti-GAPDH (Sigma-Aldrich, Vienna, Austria), and anti-β-actin (Cell Signaling Technology, Leiden, The Netherlands). Polyclonal sera were used to detect HpHtrA and CagA. Western blots were developed using HRP-coupled species-specific secondary antibodies by chemiluminescence using the Odyssey Fc imaging system (Li-Cor Biosciences, Bad Homburg, Germany). Quantification was conducted using Image Studio (Li-Cor Biosciences, Bad Homburg, Germany).

### 5.4. Real-Time PCR

Cells were lysed in TRIzol (Thermo Fisher Scientific, Vienna, Austria) and RNA was extracted according to manufacturer’s instructions. Genomic DNA was removed by DNase I (Thermo Fisher Scientific, Vienna, Austria) treatment and total RNA was used as template for cDNA synthesis with RevertAid H Minus Reverse Transcriptase (Thermo Fisher Scientific, Austria). Reverse transcription was performed according to manufacturer’s protocol with random hexamer primers (Thermo Fisher Scientific, Vienna, Austria) in a Nexus GX2 thermocycler (Eppendorf, Vienna, Austria). For quantitative real-time PCR (qPCR), equal dilutions of cDNA were used and amplified with primers that are specific for Cdh1 transcripts (forward: CTCTCACGCTGTGTCATCCA, reverse: CACCTTCCATGACAGACCCC). The qPCR was performed with TB Green Premix Ex Taq II (Takara, Paris, France) in a LightCycler 96 (Roche, Vienna, Austria). The ribosomal protein lateral stalk subunit P0 (RPLP0) was detected as loading control using specific primers (forward: CAGGTGTTCGACAATGGCAGCA, reverse: CAGACACTGGCAACATTGCGGA) and relative CDH1 expression was determined.

### 5.5. Immunofluorescence

Mucosoids were cultured for 21 days as described above. On the day of infection, the mucus was removed, and cells were infected at an MOI of 10 for 16 h. Cells were fixed with 2% PFA (Thermo Fischer, Vienna, Austria) for 20 min at 37 °C. Cells were then permeabilized using 0.2% Triton-X100 in PBS and blocked with 3% BSA (Carl Roth, Karlsruhe, Germany). Mucosoids were stained with anti-Cdh1 antibody (ab40772, Abcam, Cambridge, UK) and *H. pylori* was detected using a CagA-specific mouse serum. DNA was stained with 4′,6-diamidino-2-phenylindole (DAPI) (Carl Roth, Karlsruhe, Germany). After incubation with secondary antibodies coupled to STAR-RED or STAR580 dyes (Abberior, Göttingen, Germany), transwell filters were excised from the inserts and mounted onto glass slides using Mount Solid Antifade (Abberior, Göttingen, Germany). Image acquisition and optical z-sections by confocal laser scanning microscopy was performed on a STEDYCON unit (Abberior, Göttingen, Germany) using a Zeiss Observer Z1 platform.

### 5.6. TEER Measurements and Bacterial Transmigration

An amount of 1 × 10^5^ mucosoid-derived primary gastric cells were seeded on collagen-coated 8 µm hanging transwell filters (Sigma-Aldrich, Vienna, Austria) and cultured for three weeks in organoid medium as described above. Hanging filters were apically infected with *H. pylori* for 48 h at an MOI of 10 after mucus removal. TEER of epithelial monolayers was measured before infection and after 4, 8, and 24 h of infection using an Epithelial Volt-Ohm Meter (Millicell ERS-2, Sigma-Aldrich, Vienna, Austria) to assess the epithelial barrier integrity. During measurements, plate temperature was maintained at 37 °C. The TEER value of an empty filter was subtracted from all the readings. Bacterial transmigration was quantified in cfu assays. Briefly, medium from the lower reservoir was collected after 8 and 24 h of infection, centrifuged (3500× *g*, 5 min), resuspended in brain heart infusion broth (Carl Roth, Karlsruhe, Germany), and serial dilutions were plated in triplicates. Colony numbers were determined after 6 days of growth under microaerophilic conditions.

### 5.7. Statistics

Statistics were calculated from independent experiments as indicated. Statistical analysis was performed using GraphPad Prism 9 (GraphPad, La Jolla, CA, USA). Western blots, ELISA, and TEER experiments were evaluated using one-way ANOVA. Student’s t-test was performed for the CFU analysis of the different strains. RT-PCR analyses were analyzed using one-way ANOVA and Tuckey’s post-hoc test. Significance is indicated as non-significant (ns), * for *p* < 0.05, ** for *p* < 0.01, *** for *p* < 0.001, and **** for *p* < 0.0001.

## Figures and Tables

**Table 1:** Basic clinicopathologic characteristics of patients and their gastric sleeve specimens.

ID	Date ^1^	Gender	Age ^2^	BMI ^3^	Obese Classification ^4^	Comorbidity AH/DM-II/PAS ^5^	Medicaments ^6^	HP Eradication ^7^	Histological Diagnosis ^8^
1	2021/04	male	37.3	41.5	III	no/no/yes	No	No	Unremarkable
2	2021/04	female	21.2	58.6	III	yes/yes/yes	Yes (Metformin, Trajenta)	No	Unremarkable
3	2021/06	female	20.8	46.6	III	no/no/yes	No	No	Type C gastritis
4	2021/09	male	37.0	45.4	III	no/no/no	Yes (Pantoloc)	Yes (5)	Type C gastritis
5	2022/07	female	33.2	38.4	II	no/no/no	No	No	Type C gastritis
6	2023/10	male	44.2	50.5	III	yes/yes/no	Yes (Amlodipin, Pantoloc, Ramipril)	No	Unremarkable

^1^ Year/month of surgery. ^2^ Age at surgery in years. ^3^ BMI pre-operative [kg/m^2^]. ^4^ See: [37]. ^5^ Abbreviations: AH = arterial hypertension; DM-II = Diabetes mellitus type II; HP = *Helicobacter pylori*; PAS = previous abdominal surgery. ^6^ No, yes (drug trade name). ^7^ No, yes (month before surgery). ^8^ diagnosis of gastric sleeve specimen.

## Data Availability

The original contributions presented in the study are included in the article, further inquiries can be directed to the corresponding author.
